# Topological Surface‐Dominated Spintronic THz Emission in Topologically Nontrivial Bi_1−_ *_x_* Sb*_x_* Films

**DOI:** 10.1002/advs.202200948

**Published:** 2022-05-21

**Authors:** Hanbum Park, Seungwon Rho, Jonghoon Kim, Hyeongmun Kim, Dajung Kim, Chul Kang, Mann‐Ho Cho

**Affiliations:** ^1^ Department of Physics Yonsei University Seoul 03722 Republic of Korea; ^2^ Department of Electrical and Computer Engineering National University of Singapore Singapore 119260 Singapore; ^3^ Department of Physics Chonnam National University Gwangju 61186 Republic of Korea; ^4^ Advanced Photonics Research Institute Gwangju Institute of Science and Technology Gwangju 61005 Republic of Korea; ^5^ Department of System Semiconductor Engineering Yonsei University Seoul 03722 Republic of Korea

**Keywords:** optical spin injection, spin–charge interconversion, terahertz spectroscopy, topological phase transition, topological surface state

## Abstract

Topological materials have significant potential for spintronic applications owing to their superior spin–charge interconversion. Here, the spin‐to‐charge conversion (SCC) characteristics of epitaxial Bi_1−_ *_x_* Sb*_x_* films is investigated across the topological phase transition by spintronic terahertz (THz) spectroscopy. An unexpected, intense spintronic THz emission is observed in the topologically nontrivial semimetal Bi_1−_ *_x_* Sb*_x_* films, significantly greater than that of Pt and Bi_2_Se_3_, which indicates the potential of Bi_1−_ *_x_* Sb*_x_* for spintronic applications. More importantly, the topological surface state (TSS) is observed to significantly contribute to SCC, despite the coexistence of the bulk state, which is possible via a unique ultrafast SCC process, considering the decay process of the spin‐polarized hot electrons. This means that topological material‐based spintronic devices should be fabricated in a manner that fully utilizes the TSS, not the bulk state, to maximize their performance. The results not only provide a clue for identifying the source of the giant spin Hall angle of Bi_1−_ *_x_* Sb*_x_*, but also expand the application potential of topological materials by indicating that the optically induced spin current provides a unique method for focused‐spin injection into the TSS.

## Graphical abstract

The authors demonstrate intense spintronic terahertz emission in topologically nontrivial semimetal Bi_1−_ *_x_* Sb*_x_* films. The topological surface state is observed to significantly contribute to it, which is possible via a unique ultrafast spin‐to‐charge process. These results not only provide a clue for identifying the source of giant spin Hall angle of Bi_1−_ *_x_* Sb*_x_*, but also expand the potential for spintronic applications.

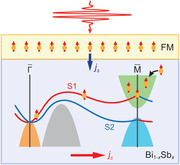

## 1 Introduction

Spin–charge interconversion has received considerable interest owing to rich spintronic phenomena and the potential for spintronic applications. The inverse spin Hall effect provides an efficient method for converting a spin current (*j*_s_) to a charge current (*j*_c_), which is described by $j_{c}=\frac{2e}{\hbar }\gamma j_{s}\times \sigma$, where *e* is the elementary charge, ℏ is the reduced Planck constant, *γ* is the spin Hall angle, and *σ* is the spin polarization. Because the spin Hall angle quantifying the interconversion efficiency is a key parameter for spintronic applications, significant efforts have been devoted to discovering materials with large spin Hall angles.^[^ ^1^, ^2^ ^]^ In principle, if the transverse spin‐polarized electrons are fully deflected to the longitudinal direction, the spin Hall angle becomes 1.^[^ ^3^ ^]^ However, a novel mechanism, called the inverse Edelstein effect, in topological materials (e.g., Bi_2_Se_3_, (BiSb)_2_Te_3_, and Bi_1−_ *_x_* Se*_x_*) makes it possible to overcome this limit effectively (*γ* > 1). ^[^ ^4^ ^–^ ^8^ ^]^

Recently, a giant spin Hall angle of 52 has been reported for Bi_0.9_Sb_0.1_ alloy,^[^ ^9^ ^]^ and a theoretical study demonstrated that the spin Hall angle can be tuned depending on the Sb concentration.^[^ ^10^ ^]^ In addition, Bi_1−_ *_x_* Sb*_x_* alloys have different topological phases depending on the Sb concentrations;^[^ ^11^, ^12^, ^13^ ^]^ for instance, they undergo a topological phase transition from the topologically trivial semimetal (*Z*_2_ =  0) to the nontrivial semimetal (*Z*_2_ = 1) at *x* ≈ 0.04 and exhibit the topological insulator phase in the range of *x*  =  0.07 − 0.23. Therefore, the topological surface state (TSS), trivial Rashba‐type surface state, and bulk state can independently contribute to the spin–charge interconversion, which makes it difficult to determine the source of the giant spin Hall angle. To develop high‐performance spintronic devices using Bi_1−_ *_x_* Sb*_x_* alloys, one must identify the source of the giant spin Hall angle. Although some researchers have attempted to clarify it,^[^ ^14^, ^15^, ^16^ ^]^ its source in Bi_1−_ *_x_* Sb*_x_* alloys with various topological phases has not been elucidated yet.

In this study, we investigated the spin‐to‐charge conversion (SCC) characteristics of epitaxial Bi_1−_ *_x_* Sb*_x_* films by spintronic terahertz (THz) spectroscopy, which is an emerging tool in the analysis of ultrafast SCC dynamics. ^[^ ^17^, ^18^, ^19^ ^]^ An ultrafast spin current was generated in the ferromagnet Co layer using an optical pump and converted to charge current in the Bi_1−_ *_x_* Sb*_x_* layer, resulting in THz radiation. Consequently, we observed a significantly stronger spintronic THz emission than those of Pt and Bi_2_Se_3_ in the topologically nontrivial semimetal Bi_1−_ *_x_* Sb*_x_* films, indicating the application potential of Bi_1−_ *_x_* Sb*_x_* as a high‐performance spintronic device and THz emitter.^[^ ^20^ ^]^ In contrast, a tight binding calculation^[^ ^10^ ^]^ and study on polycrystalline Bi_1−_ *_x_* Sb*_x_* films^[^ ^15^ ^]^ showed that SCC rapidly decreases for the nontrivial phase (*x* > 0.2). We observed that this difference caused via the contribution of the TSS in this study, despite the coexistence of the metallic bulk state, may provide a clue to identify the source of the giant spin Hall angle.

To explain the TSS‐dominated spintronic THz emission, we introduced a unique ultrafast SCC process that considers the decay process of the majority‐spin hot electrons; this is based on the phenomenon that spin‐polarized electrons are excited by the optical pump,^[^ ^21^ ^]^ unlike other spin‐injection methods. Based on this interpretation, we suggest that the optically induced spin current provides a unique method for focused‐spin injection into the TSS. Therefore, this focused‐spin injection will facilitate the investigation of the SCC characteristics of the TSS in topological materials with a reduced bulk contribution and will expand the application potential of the topological materials for spintronics.

## 2 Structural Properties of Epitaxial Bi_1−_ *_x_* Sb*_x_*

**Figure 1:**
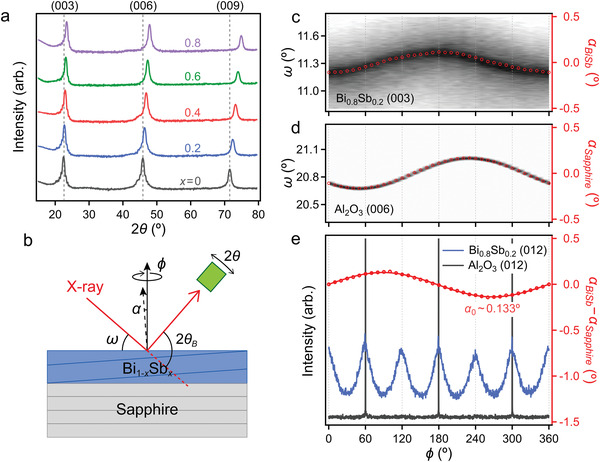
a) *θ*−2*θ* XRD spectra of the Bi_1−_ *_x_* Sb*_x_* films. b) Schematic of the experimental geometry for the *ω*−*φ* 2D scans. *ω*−*φ* 2D maps, and offset angle *α* of the c) Bi_0.8_Sb_0.2_ (003) and d) sapphire (006) planes, respectively. e) Differences in the offset angles and fitting curve of the Bi_0.8_Sb_0.2_ film with the *φ* scan spectra of the Bi_0.8_Sb_0.2_ (012) and sapphire (012) planes.

Epitaxial Bi_1−_ *_x_* Sb*_x_* films with various Sb concentrations were successfully grown on c‐plane sapphire substrates using molecular beam epitaxy. The precise Sb concentration (*x*) was determined via in situ X‐ray photoelectron spectroscopy (XPS) (Figure S1a and Note S1, Supporting Information). To characterize the crystal orientation of Bi_1−_ *_x_* Sb*_x_* films, we conducted X‐ray diffraction (XRD) measurements with various degrees of freedom. The *θ*−2*θ* XRD spectra of Bi_1−_ *_x_* Sb*_x_* films (**Figure** **1**a), exhibiting only (003) peak families in hexagonal indexing, consistently shifted to a higher angle as *x* increased. The behavior of the shift was consistent with the XPS data, indicating a homogeneous mixture of Bi and Sb (Table S1, Supporting Information). We also confirmed from Laue oscillations that the entire film was composed of a crystalline layer without an amorphous region (Figure S2 and Note S1, Supporting Information). In contrast, the Sb film (*x*  =  1.0) was polycrystalline even after postannealing, unless the Bi_1−_ *_x_* Sb*_x_* (*x* ≤ 0.9) seed layer was inserted before the growth of the Sb film. Hence, to exclude the seed layer effect, in this paper, we discuss the Bi_1−_ *_x_* Sb*_x_* films in the range of *x*  =  0 − 0.8.

Here, we note that unintentional miscut can easily occur for a sapphire substrate, resulting in crystallographic misorientation between the epitaxial film and substrate. Such a misorientation can affect the photocarrier dynamics and even become the dominant mechanism of THz radiation.^[^ ^22^, ^23^ ^]^ To verify the misorientation of the Bi_1−_ *_x_* Sb*_x_* film and substrate, we conducted *ω*−*φ* 2D scans for the Bi_1−_ *_x_* Sb*_x_* (003) and sapphire (006) lattice planes. Figure 1b shows a schematic of the experimental geometry of the *ω*−*φ* 2D scans. The offset angle (*α*) of each lattice plane as a function of sample azimuthal angle (*φ*) is given by the difference between the maximum diffraction position (*ω*) and Bragg's angle (*θ*_B_), *α* (*φ*) =  *ω*(*φ*) − *θ*_B_. Subsequently, the misorientation angle (*α*_0_) between the Bi_1−_ *_x_* Sb*_x_* film and sapphire can be determined by fitting the difference in the offset angles with *α*_BiSb_(*φ*) − *α*_Sapphire_ (*φ*) = *α*_0_ sin *φ*.

**Figure 2:**
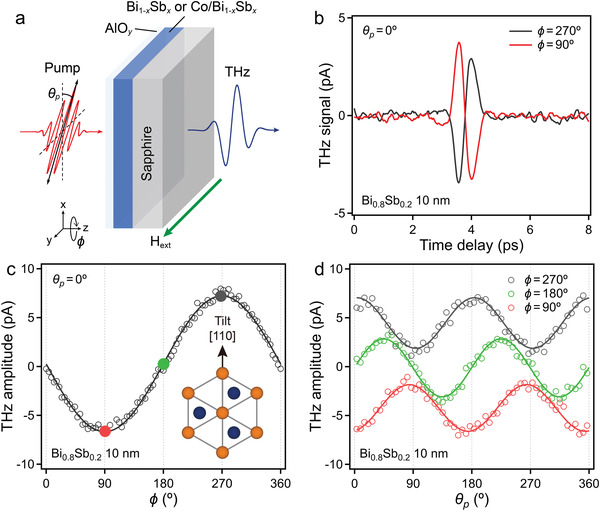
a) Schematic of the experimental geometry for THz emission measurement. b) THz emission signal of the 10 nm thick Bi_0.8_Sb_0.2_. c) Sample azimuthal angle and d) pump polarization angle dependence of the THz amplitudes for the 10 nm thick Bi_0.8_Sb_0.2_. Inset: Top view of a Bi_1−_ *_x_* Sb*_x_* bilayer.

Figure 1c,d shows the *ω*−*φ* 2D maps and offset angles of the Bi_0.8_Sb_0.2_ (003) and sapphire (006) planes, respectively. Using the *ω*−*φ* 2D maps, we obtained the difference in the offset angles and the fitting curve of *α*_0_ (Figure 1e). The sinusoidal fitting curve indicated that the *ab* plane of Bi_0.8_Sb_0.2_ was tilted with respect to the sapphire along the *φ*  =   ± 90° direction. The fitted *α*_0_ of the Bi_0.8_Sb_0.2_ film and sapphire was ≈0.133°, resulting in a change in the THz radiation mechanism. In addition, the *φ* scan spectra of the Bi_0.8_Sb_0.2_ (012) and sapphire (012) planes are shown in Figure 1e. The Bi_0.8_Sb_0.2_ (012) peaks exhibited a sixfold symmetry, indicating the existence of twin domains owing to weak van der Waals interactions,^[^ ^24^ ^]^ whereas the sapphire (012) peaks exhibited a threefold symmetry. The twin domains also affected the THz radiation, as discussed later. According to the *φ* relation between the offset angle and (012) peaks, the miscut direction of our substrates (i.e., tilt direction of the Bi_0.8_Sb_0.2_ film) was [110], as depicted in the inset of **Figure** **2**c.

## 3 THz Radiation in Bi_1−_ *_x_* Sb*_x_*

To clarify the THz radiation mechanism, we measured the THz emission of both the Bi_1−_ *_x_* Sb*_x_* and Co (5 nm)/Bi_1−_ *_x_* Sb*_x_* films. Figure 2a shows a schematic of the experimental geometry of the THz emission measurements. The Bi_1−_ *_x_* Sb*_x_* or Co/Bi_1−_ *_x_* Sb*_x_* film was exposed to a linearly polarized pump pulse (1.55 eV, ≈420 mW) at normal incidence, and only the *x*‐component of the emitted THz pulse was detected by the photoconductive antenna. Because an obtained THz signal (*S*) is a convolution of the THz field (*E*) and the system response function, it should be described using a proportional relation as *S*∝*E^x^*. The THz emission signals (*S*) of the 10 nm thick Bi_0.8_Sb_0.2_ film exhibiting the topological insulator phase were obtained in both sample azimuthal angle (*φ*) of 90° and 270°, for which the pump polarization angle (*θ*_p_) was fixed at 0° (Figure 2b).

THz radiation in Bi_1−_ *_x_* Sb*_x_* films can result from various effects, such as shift current, photo‐Dember effect, surface depletion field, and optical rectification.^[^ ^25^ ^]^ If the shift current contributes to the THz radiation, the *φ* dependence of the THz amplitude (peak‐to‐peak value of the THz signal) must exhibit a threefold symmetry for the single crystalline Bi_1−_ *_x_* Sb*_x_* because the shift current depends on the crystal symmetry.^[^ ^18^, ^23^ ^]^ However, the *φ* dependence of the THz amplitude for the 10 nm thick Bi_0.8_Sb_0.2_ exhibited a simple onefold symmetry (Figure 2c), indicating that the THz emission from the shift current was negligible in Bi_0.8_Sb_0.2_. The negligible shift current signal results from the emitted THz pulses from each twin domain being cancelled out (Figure S3 and Note S2, Supporting Information). In transmission geometry, the emitted THz pulses from the photo‐Dember effect and surface depletion field are generally not detected at normal incidence because the surge current propagates along the surface normal direction. However, since the normal direction of the *ab* plane of Bi_1−_ *_x_* Sb*_x_* was tilted with respect to the optical axis by *α*_0_, the THz pulses from the photo‐Dember effect and surface depletion field can be detected.^[^ ^26^ ^]^ As *φ* is rotated, the tilt direction also rotates, resulting in a onefold symmetric THz emission, which is consistent with the results in Figure 2c.

The *θ*_p_ dependence of THz amplitude indicated another possible source of THz radiation other than the surge current by the photo‐Dember effect and surface depletion field (Figure 2d), because the THz pulse from the surge current was independent of *θ*_p_. Optical rectification is a nonlinear effect determined by the second‐order susceptibility *χ* ^(2)^, which can contribute to THz radiation depending on the crystal symmetry as the shift current.^[^ ^27^ ^]^ Since the crystallographic misorientation changes the *χ* ^(2)^ in the tilt direction,^[^ ^28^ ^]^ the emitted THz pulse from the optical rectification exhibits a onefold symmetry on the *φ* and a twofold symmetry on the *θ*_p_. Therefore, the obtained THz signal of the Bi_1−_ *_x_* Sb*_x_* films can be described by *S*  = *A*_surge_ sin *φ* + *A*_OR_sin (*φ* + 2*θ*_p_), where *A*_surge_ and *A*_OR_ are the THz amplitudes from the surge current and optical rectification, respectively. More details of the THz signal of the Bi_1−_ *_x_* Sb*_x_* films (e.g., the fitting results and extracted THz signals from each source) are discussed in Note S2 (Supporting Information).

## 4 Spintronic THz Radiation in Co/Bi_1−_ *_x_* Sb*_x_*

**Figure 3:**
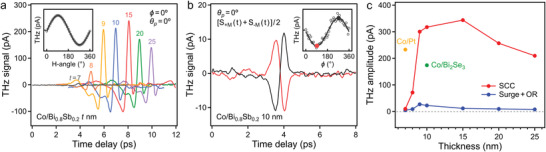
a) THz emission signals of Co/Bi_0.8_Sb_0.2_ (*t* nm). Inset: Magnetic field angle dependence of the THz amplitude for Co/Bi_0.8_Sb_0.2_ (10 nm). b) Spin‐independent THz emission signals of Co/Bi_0.8_Sb_0.2_ (10 nm). Inset: Sample azimuthal angle dependence of the spin‐independent THz amplitude. c) THz amplitudes from the SCC and other factors for Co/Bi_0.8_Sb_0.2_ as a function of the Bi_0.8_Sb_0.2_ thickness. Green and yellow dots: THz amplitudes for Co/Bi_2_Se_3_ (10 nm) and Co/Pt (7 nm), respectively.

To characterize the spintronic properties of the Bi_1−_ *_x_* Sb*_x_*, we investigated the THz emission in the Co/Bi_1−_ *_x_* Sb*_x_* heterostructures. The THz emission signals of the Co/Bi_0.8_Sb_0.2_ (7 − 25 nm) heterostructures for *φ*  =  0° and *θ*_p_ =  0° (**Figure** **3**a) were significantly larger than that of the pure Bi_0.8_Sb_0.2_ (10 nm). Moreover, the sinusoidal dependence on the external magnetic field angle (inset of Figure 3a) indicated that the intense THz signal originated from the SCC. Subsequently, we obtained the spin‐independent contribution of the THz signal given by [*S*_+M_(*t*) + *S*_−M_(*t*)]/2, where *S*_+M_(*t*) and *S*_−M_(*t*) are the THz signals for the opposite magnetization directions, ± M (i.e., the magnetic field angle is 90° and 270°). Figure 3b shows the spin‐independent THz emission signal of Co/Bi_0.8_Sb_0.2_ (10 nm), the *φ* dependence of which was identical to the 10 nm thick Bi_0.8_Sb_0.2_ (inset of Figure 3b). Since the Co/Bi_1−_ *_x_* Sb*_x_* films had different interfaces from the Bi_1−_ *_x_* Sb*_x_* films, the surface depletion field was modified, resulting in the enhancement of the spin‐independent signal of the Co/Bi_1−_ *_x_* Sb*_x_* films. The electrons injected from the Co may have also contributed to the spin‐independent signal.

We plotted the THz amplitude extracted from the SCC and other factors (surge current + optical rectification) as a function of the Bi_0.8_Sb_0.2_ thickness (Figure 3c). The maximum THz amplitude for Co/Bi_0.8_Sb_0.2_ was significantly larger than those of Co/Bi_2_Se_3_ and Co/Pt, as indicated by green and yellow dots, respectively, in Figure 3c, owing to the large spin Hall angle; the THz waveforms of Co/Bi_2_Se_3_ and Co/Pt are shown in Figure S7a (Supporting Information). The spintronic THz field is given by^[^ ^17^ ^]^

(1) $$E\left(\omega \right)=Z\left(\omega \right)\frac{2e}{\hbar }\sum_{i}\int \mathrm{dz}\gamma_{i}\left(z\right)j_{s}\left(z,\omega \right)$$

where *Z*(*ω*) is the impedance of the sample, *γ*_i_(*z*) is the spin Hall angle, and *j*_s_(*z*,*ω*) is the spin current. In Bi_1−_ *_x_* Sb*_x_* films, the surface and bulk states can independently contribute to the SCC with different spin Hall angles. If the inverse spin Hall effect from the bulk state is a principal source of the spintronic THz radiation, the obtained THz signal should be described by the spin‐diffusion model,^[^ ^17^, ^18^ ^]^ $S\propto Z\frac{A}{d_{\mathrm{FM}}+t}\gamma_{\mathrm{bulk}}\lambda_{s}\tanh\frac{t}{2\lambda_{s}}$, where *A* is the absorptance, *d*_FM_ is the Co thickness, *t* is the Bi_1−_ *_x_* Sb*_x_* thickness, and *λ*_s_ is the spin‐diffusion length. However, our experimental data shown in Figure 3c could not be fitted with this hyperbolic tangent function (more details are discussed in Figure S9 and Note S3, Supporting Information) and indicated unconventional thickness dependence, which is a distinct result from sputter‐grown polycrystalline Bi_1−_ *_x_* Sb*_x_* films that exhibit bulk‐dominated charge‐to‐spin conversion.^[^ ^15^ ^]^

Our experimental data were rather similar to those of epitaxial Bi_2_Se_3_ films that exhibit surface‐dominated SCC.^[^ ^18^, ^19^, ^29^ ^]^ In the ultrathin regime (often <  6 nm), the intersurface coupling of a topological insulator creates a gap at the Dirac point and reduces the spin polarization of the TSS, resulting in a critical decrease in the spin Hall angle.^[^ ^19^ ^]^ The intersurface coupling of an 8 nm thick Bi_1−_ *_x_* Sb*_x_* is not sufficiently strong to create a gap at the $\bar{\Gamma }$ point, but the coupling elsewhere in the *k*‐space can be significant owing to a large tunneling probability.^[^ ^30^, ^31^, ^32^ ^]^ In contrast to the Bi_2_Se_3_, because the complicated surface state of Bi_1−_ *_x_* Sb*_x_* alloys generates many anisotropic electron and hole pockets, the spin polarization of the surface state away from the $\bar{\Gamma }$ point should be considered to investigate the SCC. When approaching the $\bar{M}$ point, the surface penetration depth and coupling strength increase, resulting in a loss in the spin polarization even in the 8 nm thick Sb film.^[^ ^31^, ^32^ ^]^ In addition, under 7 nm, the emerging pseudocubic phase has a different surface‐spin configuration to hexagonal the phase (Figure S2 and Note S1, Supporting Information), which can reduce the spin polarization of the (003) surface.^[^ ^33^ ^]^ Therefore, if the surface state is a principal source of the spintronic THz radiation, the abrupt decrease in the spintronic THz signal under 8 nm possibly results from the intersurface coupling and multiphase structure.

Another possibility is that the surface and bulk states simultaneously contribute to the SCC with opposite directions. Because the surface‐induced charge current is more rapidly saturated with thickness than the bulk owing to the short spin‐diffusion length, the total charge current can be cancelled out at a certain thickness. A similar phenomenon was observed in Bi films; the surface and bulk state contribute to the SCC with an opposite spin Hall angle in a crystalline Bi/Py heterostructure,^[^ ^34^ ^]^ whereas only the bulk state contributes to the SCC in amorphous Bi/Py.^[^ ^35^ ^]^ Based on this consideration, the spintronic THz amplitude must exhibit a negative value under 7 nm. However, this expectation is not consistent with the observation that the SCC amplitude under 7 nm was negligible. More details for the validity of the possible considerations are provided in Note S3 (Supporting Information).

**Figure 4:**
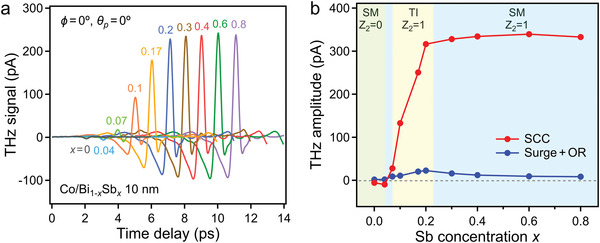
a) THz emission signals of Co/Bi_1−_ *_x_* Sb*_x_* (10 nm). b) THz amplitudes from the SCC and other factors of Co/Bi_1−_ *_x_* Sb*_x_* (10 nm) across the topological phase transition: trivial semimetal (SM) to topological insulator (TI) and nontrivial semimetal.

To identify the source of the intense spintronic THz emission in Co/Bi_0.8_Sb_0.2_, we investigated the evolution of spintronic THz emission across the topological phase transition. **Figure** **4**a shows the THz emission signals of the Co/Bi_1−_ *_x_* Sb*_x_* (10 nm) heterostructures for *φ*  =  0° and *θ*_p_ =  0°. As conducted for Co/Bi_0.8_Sb_0.2_ (*t* nm), we extracted the THz amplitude from the SCC and another factors (surge current + optical rectification) for Co/Bi_1−_ *_x_* Sb*_x_* (10 nm) (Figure 4b). According to the spintronic THz amplitude, three distinct regimes were realized. For *x* ≤ 0.04, Bi_1−_ *_x_* Sb*_x_* exhibited the trivial semimetal phase (*Z*_2_ =  0) and negligible and negative THz amplitudes. Because the pure Co also exhibits negative spintronic THz emission with comparable amplitude, the THz emission of Bi_1−_ *_x_* Sb*_x_* may mostly result from the pure Co in this regime. In this regime, the characteristics of the Bi_1−_ *_x_* Sb*_x_* are almost similar to those of Bi. Although Bi has strong spin–orbit coupling and large spin Hall conductivity from the bulk state,^[^ ^10^ ^]^ many studies reported a negligible SCC similar to our results.^[^ ^3^, ^36^, ^37^ ^]^

For 0.04 < *x* ≤ 0.2, in which Bi_1−_ *_x_* Sb*_x_* transitioned to the topological insulator phase (*Z*_2_ =  1) with the emergence of the TSS, the spintronic THz amplitude rapidly increased as *x* increased up to 0.2. Based on Equation (1), because the impedance decreased as *x* increased (Figure S9 and Note S3, Supporting Information), the increasing THz amplitude was caused by enhancement in the SCC efficiency (i.e., spin Hall angle). Finally, for *x* > 0.2, in which the Bi_1−_ *_x_* Sb*_x_* transitioned to the topological semimetal phase (*Z*_2_ =  1), the spintronic THz amplitude was saturated. This was a completely opposite result from the tight binding calculation^[^ ^8^ ^]^ and the sputter‐grown polycrystalline Bi_1−_ *_x_* Sb*_x_* films,^[^ ^15^ ^]^ which indicated that the SCC efficiency decreases as *x* increases and emphasized the contribution of the bulk state to the SCC. Because Bi_1−_ *_x_* Sb*_x_* (*x* ≥ 0.2) has the TSS even in the semimetal phase owing to its negative indirect gap,^[^ ^11^ ^]^ our experimental results implied that the TSS, not the bulk state, has an important function in SCC.

## 5 Ultrafast SCC Process through TSS

**Figure 5:**
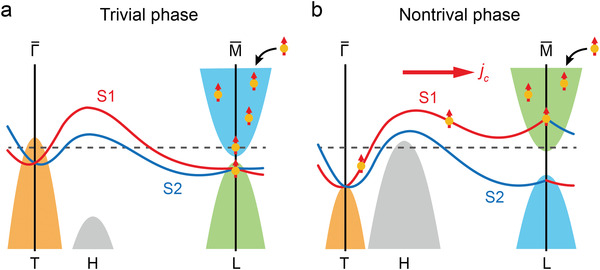
Band structure of Bi_1−_ *_x_* Sb*_x_* and ultrafast SCC mechanism of the majority‐spin hot electrons for a) trivial and b) nontrivial phases.

Subsequently, based on the obtained data, we can now suggest the TSS contribution mechanism to the spintronic THz emission. For the spintronic THz emission measurements, Co and Bi_1−_ *_x_* Sb*_x_* were both pumped using the optical pump pulse (1.55 eV); hence, the spin‐polarized electrons in the Co were excited. Because the majority‐spin hot electrons have a longer decay time than the spin injection time,^[^ ^18^, ^21^ ^]^ the hot electrons recombined with holes in the Bi_1−_ *_x_* Sb*_x_* layer. **Figure** **5** shows the band structure of Bi_1−_ *_x_* Sb*_x_* and ultrafast SCC mechanism of the majority‐spin hot electrons for trivial and nontrivial phases.^[^ ^12^, ^13^ ^]^ For the trivial phase, two trivial surface bands with opposite spin polarization dispersed along $\bar{\Gamma M}$ within the bulk bandgap and connected the bulk *T* and *L* valence bands, satisfying Kramers degeneracy at $\bar{\Gamma }$ and $\bar{M}$. Because the direct bandgap at the *L* point was very small (e.g., ≈14 meV for *x*  =  0),^[^ ^13^ ^]^ most majority‐spin hot electrons decayed directly from the *L* conduction band to the *L* valence band.

For the nontrivial phase, the S1 surface band became topological owing to the inversion of the bulk *L* band, whereas the S2 surface band remained trivial. In addition, because the direct bandgap at the *L* point increased with Sb concentration (e.g., ≥ 100 meV for *x*  =  0.21),^[^ ^13^ ^]^ most majority‐spin hot electrons decayed through the S1 surface band connecting the bulk *T* valence and *L* conduction bands. Subsequently, the decay of the majority‐spin hot electrons occurred in one direction satisfying the selection rule, resulting in a unidirectional charge current (i.e., SCC) and THz radiation. Furthermore, the population of the S1 surface band by majority‐spin hot electrons would have lasted for a long time owing to the long lifetime of the TSS,^[^ ^38^, ^39^ ^]^ thus enhancing the contribution of the S1 surface band. This is supported by Bi_1−_ *_x_* Sb*_x_* and Bi_2_Se_3_ having the same sign of the spin Hall angle (Figure S7a and Note S2, Supporting Information) because the spin‐polarization direction of the S1 surface band was identical to that of the TSS in Bi_2_Se_3_. Thus, although the trivial surface state was also spin polarized in Bi_1−_ *_x_* Sb*_x_*, the TSS had a more critical role in the spintronic THz emission. If Bi_1−_ *_x_* Sb*_x_* contains many defects (e.g., sputter‐grown polycrystalline film), more majority‐spin hot electrons decay directly at the *L* point through the defect state, suppressing the contribution of the TSS.

## 6 Conclusion

We investigated the evolution of spintronic THz emission in Bi_1−_ *_x_* Sb*_x_* films as a function of the thickness and Sb concentration. Through a detailed review of possible scenarios, we observed that the intense THz signal in topologically nontrivial Bi_1−_ *_x_* Sb*_x_* films results from the unique ultrafast SCC process through the TSS. This unique ultrafast SCC process may also occur with other optical spin‐injection methods, such as using circularly‐polarized light without a ferromagnetic layer.^[^ ^40^, ^41^, ^42^ ^]^ Therefore, when investigating the SCC of topological materials via optical spin‐injection methods, it should be interpreted in terms of the ultrafast SCC (i.e., considering the decay process of the spin‐polarized hot electrons). Despite the significant potential of Bi_1−_ *_x_* Sb*_x_* in spintronic applications, the source of the giant spin Hall angle has been unclear. Our results clarify the role of the TSS in the ultrafast SCC of the Bi_1−_ *_x_* Sb*_x_*, which may advance the development of high‐performance spintronic devices and THz emitters.

Although the approach through electrical methods (e.g., ferromagnetic resonance) is more popular when investigating SCC, the optical approach is a practical and very effective method for material and process verification. Through this study, we successfully extracted factors that are complicated to increase the SCC efficiency in the Bi_1−_ *_x_* Sb*_x_* thin‐film system. These results can facilitate a clear direction on which materials to develop to improve the SCC efficiency for spintronic devices. In particular, appropriate strategies for material synthesis and device fabrication should be selected in a manner that fully utilizes the TSS, not the bulk state.

## Conflict of Interest

The authors declare no conflict of interest.

## Supporting information

Supporting Information

## Data Availability

The data that support the findings of this study are available from the corresponding author upon reasonable request.
