# Supplementary material for: Topological Surface‐Dominated Spintronic THz Emission in Topologically Nontrivial Bi1− x Sb x Films
Source: Adv Sci (Weinh). 2022 May 21;9(21):2200948. doi: 10.1002/advs.202200948 (PMC9313944; doi:10.1002/advs.202200948)
Supplement: Supplementary file 1 — Supporting Information [file ADVS-9-2200948-s001.pdf]

ADVANCED SCIENCE
Open Access

Supporting Information

for *Adv. Sci.*, DOI 10.1002/advs.202200948

Topological Surface-Dominated Spintronic THz Emission in Topologically Nontrivial $Bi_{1-x}Sb_x$ Films

Hanbum Park, Seungwon Rho, Jonghoon Kim, Hyeongmun Kim, Dajung Kim, Chul Kang and Mann-Ho Cho**

Supporting Information

Topological Surface-Dominated Spintronic THz Emission in Topologically Nontrivial $Bi_{1-x}Sb_x$ Films

Hanbum Park, Seungwon Rho, Jonghoon Kim, Hyeongmun Kim, Dajung Kim, Chul Kang, and Mann-Ho Cho**

Supplementary Note 1

When Bi and Sb were deposited at temperatures higher than room temperature, the grown $Bi_{1-x}Sb_x$ film had a textured structure, resulting in a large surface roughness. Because a smooth Co/$Bi_{1-x}Sb_x$ interface is necessary to preserve the surface spin properties and study the role of the surface state in spin−charge conversion, we deposited the Bi and Sb at room temperature. Accordingly, double-sided polished c-plane (0001) sapphire substrates were first degassed at 600 °C for 1 h under a pressure of < 1 × Pa. After cooling for > 6 h to reach room temperature, Bi and Sb were simultaneously deposited on the sapphire substrates from Knudsen cells. The Sb concentration (x) was determined by in-situ X-ray photoelectron spectroscopy (XPS) and was subtly modulated by controlling the evaporation rate of each Bi and Sb atom (**Figure S1a** and **Table S1**). A low deposition rate was maintained at ~1.7 Å /min to obtain a smooth surface.

The as-grown $Bi_{1-x}Sb_x$ films were composed of a mixture of the hexagonal (003) and pseudocubic (012) phases, which were determined via X-ray diffraction (XRD) measurements (**Figure S2a**). The as-grown multiphase $Bi_{1-x}Sb_x$ films were subsequently annealed to improve the crystallinity at 100−200 °C depending on the Sb concentration (**Figure S1b**). Our post-annealing condition was selected such as to obtain a smooth surface and a single phase based on the phase diagram of $Bi_{1-x}Sb_x$ adapted from the theoretical calculation.[1] After post-annealing, they became highly (003)-oriented hexagonal $Bi_{1-x}Sb_x$ films with an in-plane crystal order. However, the pseudocubic phase was still under 7 nm, which was similar to that grown on the Si (111) substrate.[2] The AlO_y capping layer and 5-nm-thick Co film were grown using electron-beam evaporation. The entire growth process was performed without air exposure to avoid contamination (e.g., oxidation); the terahertz (THz) signal was reduced by approximately 70% when the sample was exposed to the air before the growth of the Co film.

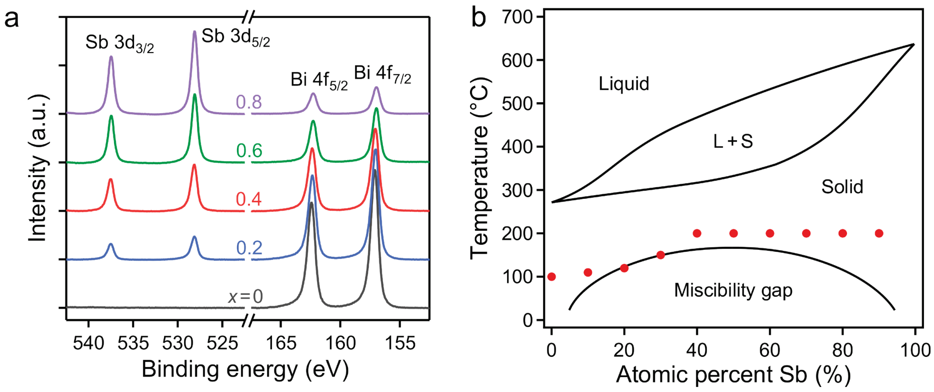

Figure S1. (a) XPS spectra of 10-nm-thick $Bi_{1-x}Sb_x$ films. (b) Post-annealing temperatures (red dot) and phase diagram of $Bi_{1-x}Sb_x$ (solid line) adapted from theoretical calculation.[1]

Table S1. Sb concentration x determined via XPS and XRD using Vegard's Law

Sample	Sb concentration x (from XPS)	Lattice parameter c (Å)	Sb concentration x (from Vegard's Law)
Bi	0	11.876	0
$Bi_{0.8}Sb_{0.2}$	0.199	11.752	0.210
$Bi_{0.6}Sb_{0.4}$	0.395	11.640	0.399
$Bi_{0.5}Sb_{0.5}$	0.496	11.581	0.5
$Bi_{0.4}Sb_{0.6}$	0.598	11.522	0.567
$Bi_{0.2}Sb_{0.8}$	0.796	11.404	0.782

The Sb concentration was reconfirmed using lattice parameters obtained from the (003) peak position with Vegard's law, which assumes that the lattice parameter of an alloy is given by a simple linear interpolation between the lattice parameters of each component. We used the lattice parameters of Bi and $Bi_{0.5}Sb_{0.5}$ as standards because the Sb film was polycrystalline. The estimated Sb concentrations are listed in **Table S1** and were mostly consistent with the XPS data. In addition, Laue oscillations of the (003) peak originating from the finite thickness of the crystalline layer were observed and became distinct as the $Bi_{1-x}Sb_x$ thickness increased (**Figure S2**). Using the Laue oscillations, the crystalline film thickness (d) can be obtained by

$$d = \frac{\lambda}{2(\sin\theta_{i+1} - \sin\theta_i)},$$

where θ_{i+1} and θ_i are adjacent maxima of the oscillations, and λ is the X-ray wavelength. Accordingly, we fit the XRD spectra of the 20-nm-thick $Bi_{0.8}Sb_{0.2}$ film with a pseudo-Voight function to obtain the first and second maxima positions (**Figure S2b**). As a result, the

obtained crystalline thickness was ~20.0 nm, which was equal to the film thickness; this meant that the entire film was composed of the crystalline layer without an amorphous region.

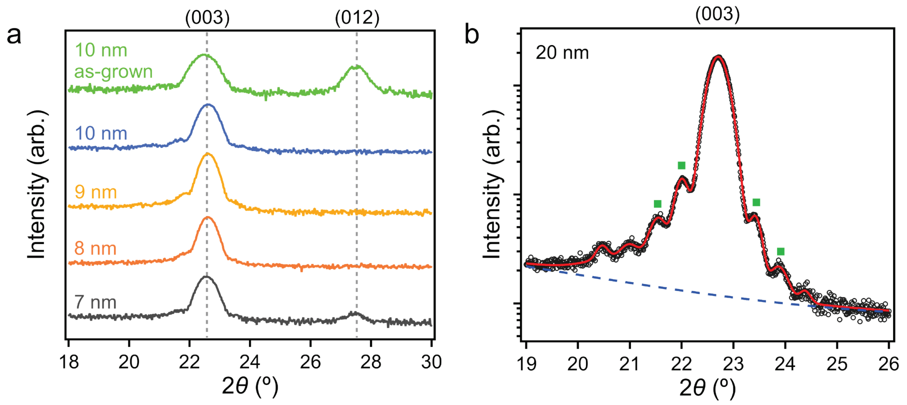

Figure S2. $\theta-2\theta$ XRD spectra of $Bi_{0.8}Sb_{0.2}$ films for (a) 7–10 nm and (b) 20 nm with Laue oscillations.

The in-plane ferromagnetic behavior of Co (5 nm)/$Bi_{0.8}Sb_{0.2}$ (10 nm) was confirmed by a vibrating sample magnetometer (VSM) (**Figure S3**). The saturated magnetization density of our sample was very similar with the reported value of the e-beam evaporated Co film.[3] The externally applied 1200 G magnetic field was sufficiently strong to saturate the sample magnetization during THz measurements.

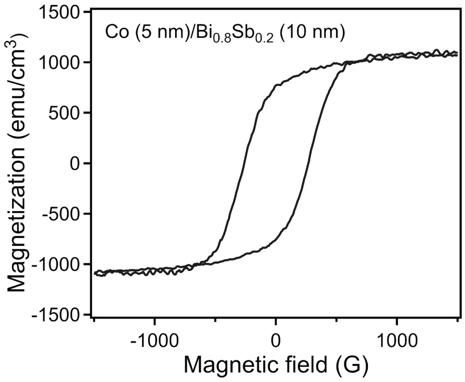

Figure S3. In-plane ferromagnetic hysteresis of Co (5 nm)/$Bi_{0.8}Sb_{0.2}$ (10 nm).

Supplementary Note 2

A standard THz time-domain spectroscopy setup was employed to measure the time-domain THz waveforms. To radiate the THz pulses, a femtosecond laser (80 MHz repetition rate, 1.55 eV, and 100 fs pulse width) was illuminated to the samples. A 300-mm bi-convex lens was used with a focal diameter of approximately 100 μm on the sample to focus the

pump pulse. A λ/2 waveplate was used to control the pump polarization. The emitted THz pulse was detected using a 5-μm dipole gap photoconductive antenna on a low-temperature grown GaAs substrate with a 5-mW fs-laser power. During the measurements, the sample saturated magnetizations were maintained by applying an external magnetic field of 1200 G.

In transmission geometry, the shift current is frequently a principal source of THz radiation via a linearly polarized pump in topological insulators.[4,5] A real-space change in the electron distribution between the valence and conduction bands generates a shift current along the atomic bonds. Based on the notation of sample azimuthal angle (ϕ) used in the XRD measurements, we depict the top view of a $Bi_{1-x}Sb_x$ bilayer for twin domains, proven to exist using a ϕ scan, with two different pump polarizations in **Figure S4**. Whereas the inversion symmetry was preserved for the blue polarization ($\theta_p = 90°$), it was broken at surface for the red polarization ($\theta_p = 0°$), resulting in the directional shift current in each twin domain. Accordingly, the possible THz signal from the shift current can be described by

$$E^x \propto S = A_{shift} \sin\left(3\phi + 2\theta_p + \pi/2\right),$$

where A_{shift} is the THz amplitude from the shift current. However, as shown in **Figure 2c** and **2d,** we could not observe any contribution from the shift current. This is because the directions of the shift current from each twin domain were opposite, and hence it was cancelled out.

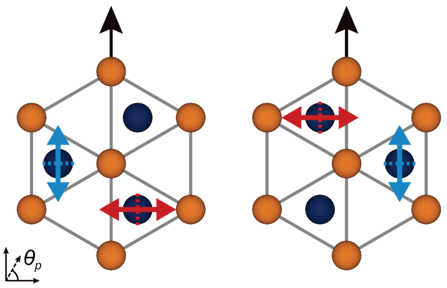

Figure S4. Top view of a $Bi_{1-x}Sb_x$ bilayer for twin domains with two different pump polarizations.

The other possible sources of THz radiation in the $Bi_{1-x}Sb_x$ films are the photo-Dember effect, the surface depletion field, and optical rectification.[5] The significant difference between the electron and hole mobility induces a charge dipole after photoexcitation, which is the called photo-Dember effect. Accordingly, the photo-Dember effect and surface depletion field both induce photocurrent surge along the surface normal direction. However, we could not identify which one is the principal source of the surge current in our experimental

variation. In non-centrosymmetric materials, the second-order nonlinear optical effect can exist and result in optical rectification. Although the crystal structure of the $Bi_{1-x}Sb_x$ is inversion symmetric, the crystallographic misorientation between the $Bi_{1-x}Sb_x$ film and the substrate enables the nonlinear optical effect to exist. The charge polarization induced by the optical rectification is given by $P^{(2)} = 2\varepsilon_0\chi^{OR}|E|^2$, where ε_0 is the vacuum permittivity, χ^{OR} is the second-order nonlinear optical susceptibility relevant to the optical rectification, and E is the electric field of the pump pulse.[6] Since the tilt direction is [110], the induced polarization is also in the [110] direction, resulting in a one-fold symmetric THz emission on the ϕ with a two-fold on the θ_p.

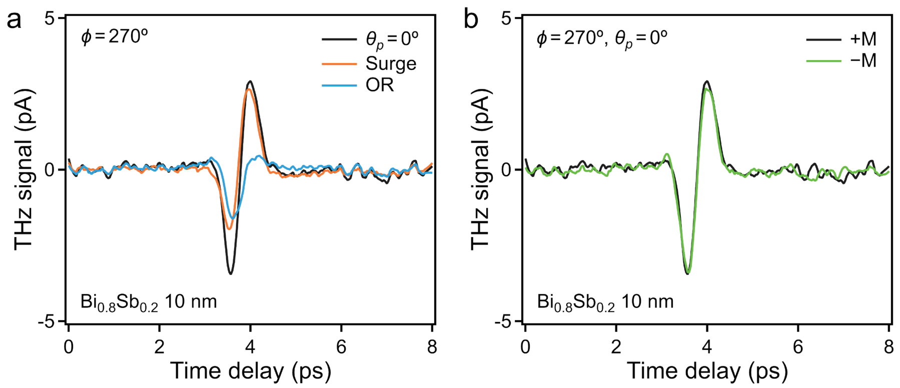

Figure S5. THz emission signal of the 10-nm-thick $Bi_{0.8}Sb_{0.2}$ (a) from the surge current and optical rectification and (b) for opposite magnetization directions, $\pm M$.

Consequently, as discussed in the main manuscript, the obtained THz signal from the surge current and optical rectification can be described by

$$S = A_{surge}\sin\phi + A_{OR}\sin(\phi + 2\theta_p),$$

where A_{surge} and A_{OR} are the THz amplitude from the surge current and optical rectification, respectively. Subsequently, we can extract the THz signals from each contribution using

$$S_{surge} = [S(\phi = 270°, \theta_p = 0°) + S(\phi = 270°, \theta_p = 90°)]/2$$

$$S_{OR} = [S(\phi = 270°, \theta_p = 0°) - S(\phi = 270°, \theta_p = 90°)]/2.$$

We plotted extracted THz signals of the 10-nm-thick $Bi_{0.8}Sb_{0.2}$ from the surge current and optical rectification (**Figure S5a**). The THz amplitude from the surge current was larger than the optical rectification for the 10-nm-thick $Bi_{0.8}Sb_{0.2}$, but it can be varied depending on the $Bi_{1-x}Sb_x$ thickness and Sb concentration.

In addition, because the THz signal from the surge current and optical rectification is spin-independent, it is identical for the opposite magnetization directions, $\pm M$ (**Figure S5b**).

Therefore, we can also extract the spin-independent and -dependent contributions in the THz signal of the Co/$Bi_{1-x}Sb_x$ using

$$S_{indep} = [S_{+M}(t) + S_{-M}(t)]/2,$$
$$S_{dep} = [S_{+M}(t) - S_{-M}(t)]/2,$$

where $S_{+M}(t)$ and $S_{-M}(t)$ are the THz signal for $\pm M$. The spin-dependent signal results from the spin-to-charge conversion (SCC) in the $Bi_{1-x}Sb_x$ layer. **Figure S6a** shows the THz emission signals of the Co/$Bi_{0.8}Sb_{0.2}$ (10 nm) for $\pm M$, which exhibited opposite polarities as expected. The ϕ dependence of the THz amplitude exhibited a one-fold symmetry independent of the magnetization direction (**Figure S6b**), which resulted from the surge current and optical rectification as the 10-nm-thick $Bi_{0.8}Sb_{0.2}$.

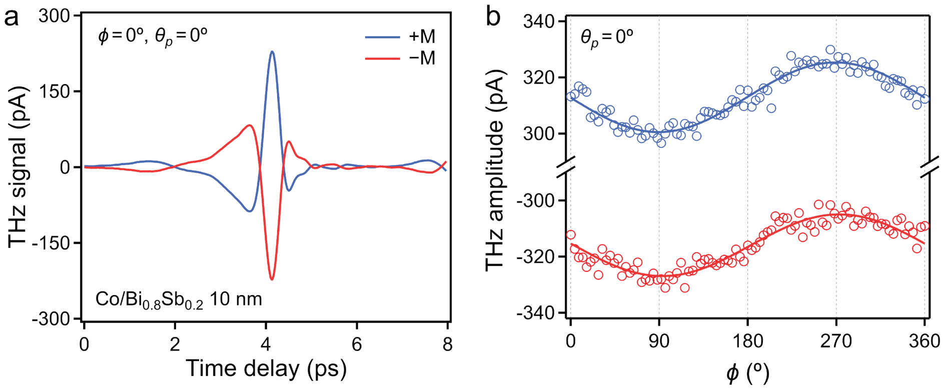

Figure S6. (a) THz emission signal and (b) sample azimuthal angle dependence of the Co/$Bi_{0.8}Sb_{0.2}$ (10 nm) for opposite magnetization directions, $\pm M$.

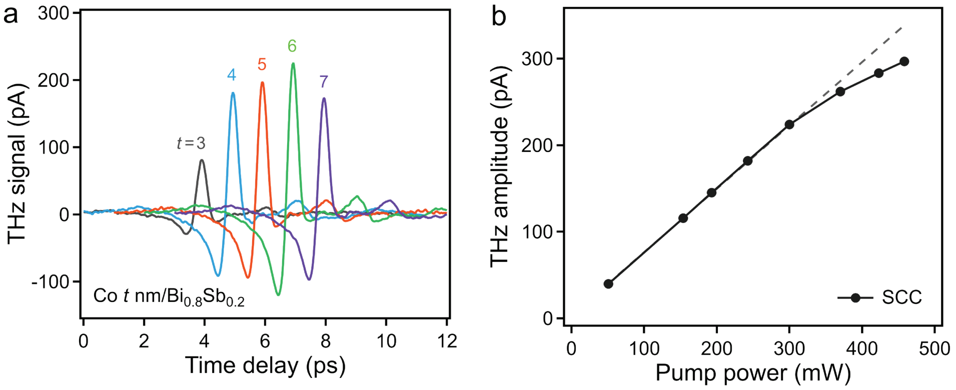

Figure S7. (a) THz emission signals of Co (t nm)/$Bi_{0.8}Sb_{0.2}$ (10 nm). (b) Pump-power dependence of the spintronic THz amplitude for Co (5 nm)/$Bi_{0.8}Sb_{0.2}$ (10 nm).

Figure S7a shows the THz signals of Co (t nm)/$Bi_{0.8}Sb_{0.2}$ (10 nm) as a function of the Co thickness. The THz amplitude increased as the Co thickness increased up to 6 nm, and then,

the amplitude decreased.[7] **Figure S7b** shows the spintronic THz amplitude of Co (5 nm)/$Bi_{0.8}Sb_{0.2}$ (10 nm) as a function of the pump power. The THz amplitude was proportional to the pump power, but it exhibited saturation behavior in the high-power region, as reported in many studies.[4,8,9]

We compared the THz signal of Co/$Bi_{0.8}Sb_{0.2}$ to that of other materials under identical experimental setup and conditions. The THz signal of Co/$Bi_{0.8}Sb_{0.2}$ exhibited the same polarity as that of Co/Bi_2Se_3 and Co/Pt, indicating the same sign of the spin Hall angle (**Figure S8a**). Whereas the THz amplitude of Co/$Bi_{0.8}Sb_{0.2}$ reached a maximum value at 15 nm, those of Co/Bi_2Se_3 and Co/Pt reached maximum values at 10 nm and 7 nm, respectively.[4,7] Consequently, the maximum THz amplitude for Co/$Bi_{0.8}Sb_{0.2}$ (344 pA at 15 nm) was significantly larger than those for Co/Bi_2Se_3 (174 pA at 10 nm) and Co/Pt (233 pA at 7 nm) owing to the large spin Hall angle.[4,10]

In addition, we compared the THz signal of Co/$Bi_{0.8}Sb_{0.2}$ with that of a standard THz crystal, 2-mm-thick ZnTe (110). Although Co/$Bi_{0.8}Sb_{0.2}$ exhibited the strongest spintronic THz emission, its THz signal was much weaker than that of ZnTe (**Figure S8b**). To obtain a comparable THz amplitude with that of the standard THz crystal, a fabrication strategy such as use of a trilayer structure is required.[10] Meanwhile, a characteristic change in Fourier-transformed THz spectra was observed (inset of Figure S8b). The THz spectrum from SCC was shifted to a lower frequency compared to that of ZnTe; the normalized THz spectra of Co/Bi_2Se_3, Co/Pt, and Co/$Bi_{1-x}Sb_x$ were similar owing to the same origin, i.e., SCC.

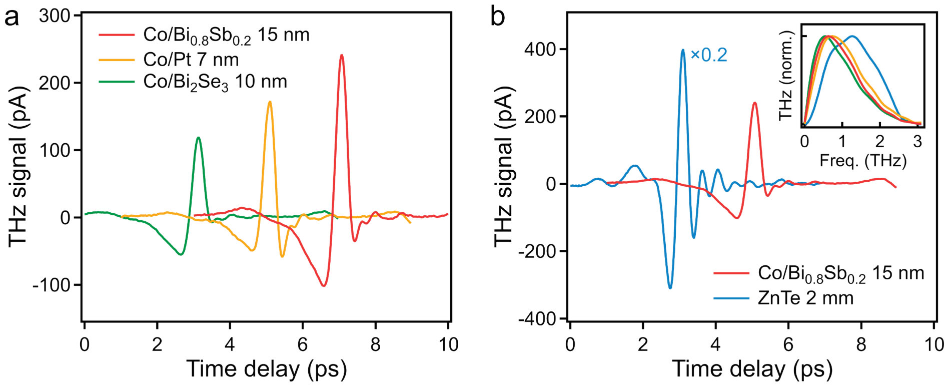

Figure S8. (a) THz emission signals of Co/Bi_2Se_3 (10 nm), Co/Pt (7 nm), and Co/$Bi_{0.8}Sb_{0.2}$ (15 nm). (b) THz emission signals of ZnTe (2 mm) (× 0.2) and Co/$Bi_{0.8}Sb_{0.2}$ (15 nm). Inset: Normalized Fourier spectra of ZnTe, Co/Bi_2Se_3, Co/Pt, and Co/$Bi_{0.8}Sb_{0.2}$.

Supplementary Note 3

Based on the spin diffusion model, the spin current at distance z from the interface is given by[11]

$$j_s(z) = j_s(0)\frac{\sinh\left[(z-t)/\lambda_{sf}\right]}{\sinh\left(t/\lambda_{sf}\right)},$$

where t is the $Bi_{1-x}Sb_x$ thickness, and λ_{sf} is the spin diffusion length. The initial spin current density $j_s(0)$ is proportional to the absorbed energy density of the pump pulse, described by $A/(d_{FM}+t)$, where A is the absorptance of the sample and d_{FM} is the Co thickness. Thus, if we assume that the inverse spin Hall effect from the bulk state is a principal source of the spintronic THz radiation, the obtained THz signal should be described by[4,10]

$$\mathrm{S}(\omega) \propto \mathrm{Z}(\omega)\frac{A}{d_{FM}+t}\gamma_{bulk}\lambda_s \tanh\frac{t}{2\lambda_s},$$

Where $\mathrm{Z}(\omega)$ is the impedance of the sample, and γ_{bulk} is the spin Hall angle of the bulk state.

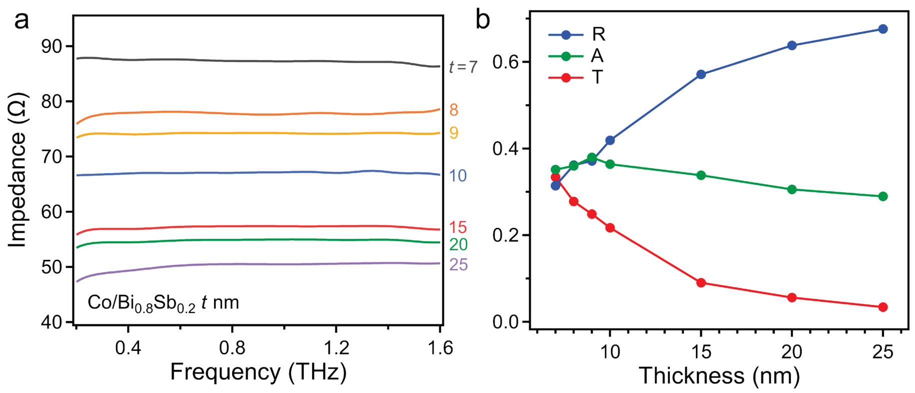

Figure S9. (a) Impedance of the $Co/Bi_{0.8}Sb_{0.2}$ (t nm). (b) Reflectance, absorptance, and transmittance of the $Co/Bi_{0.8}Sb_{0.2}$ as a function of the $Bi_{0.8}Sb_{0.2}$ thickness.

Accordingly, we measured the THz transmittance to obtain the impedance of the $Co/Bi_{0.8}Sb_{0.2}$ (t nm). The measured THz transmittance $T(\omega)$ was converted into the optical impedance $\mathrm{Z}(\omega)$ using the Tinkham formula, $T(\omega) = \left[Z(\omega)\frac{1+n_s}{Z_0}\right]^2$, where Z_0 is the vacuum impedance, and n_s is the refractive index of the substrate.[8] The obtained $\mathrm{Z}(\omega)$ of the $Co/Bi_{0.8}Sb_{0.2}$ (t nm) was almost constant within the observed frequency range (**Figure S9a**). In addition, we measured the power of the transmitted and reflected pump pulse using a beam splitter to obtain the absorptance, given by $A = 1 - T - R$, where T is the transmittance, and R is the reflectance (**Figure S9b**).

With the results of the impedance and absorptance, we attempted to fit the spintronic THz amplitude as a function of the $Bi_{0.8}Sb_{0.2}$ thickness (7–25 nm) with the spin diffusion model. However, our experimental data could not be fitted with the hyperbolic tangent function that should cross the $(0, 0)$ point (**Figure S10**). To apply the spin diffusion model, we introduced a dead layer (amorphous region) in the $Bi_{0.8}Sb_{0.2}$, similar to other studies[4,12] and modified the equation as

$$S(\omega) \propto Z(\omega) \frac{A}{d_{FM} + t} \gamma_{bulk} \lambda_s \tanh \frac{t - t_d}{2\lambda_s},$$

where t_d is the dead-layer thickness. Thus, the spin-diffusion model with the dead layer seemed to fit the experimental data well and yielded fitting values of λ_s ~2.8 nm and t_d ~7.1 nm. However, such a large t_d value is unreasonable based on the XRD results; the crystalline $Bi_{0.8}Sb_{0.2}$ film was successfully grown even under 7 nm although the pseudocubic phase slightly remained, and the entire of the 20-nm-thick $Bi_{0.8}Sb_{0.2}$ film was composed of the crystalline layer (**Supplemental Note 1**). In addition, as indicated in thickness-dependent studies of Bi and $Bi_{1-x}Sb_x$,[13–15] the multiphase consisting of the hexagonal and pseudocubic phases does not affect the SCC of the bulk state (i.e., inverse spin Hall effect). Therefore, we concluded that the inverse spin Hall effect from the bulk state is not the principal source of the large spintronic THz emission in $Bi_{1-x}Sb_x$.

As discussed in the main manuscript, another possibility is that the surface and bulk states simultaneously contribute to the SCC with opposite directions: positive for the bulk state and negative for the surface state. Based on this consideration, the spintronic THz amplitude must exhibit a negative value under 7 nm owing to the negative spintronic THz emission from the surface state. However, the SCC amplitude of the $Co/Bi_{0.8}Sb_{0.2}$ (4 nm) was negligible, which excluded the possibility of this consideration (**Figure S10**).

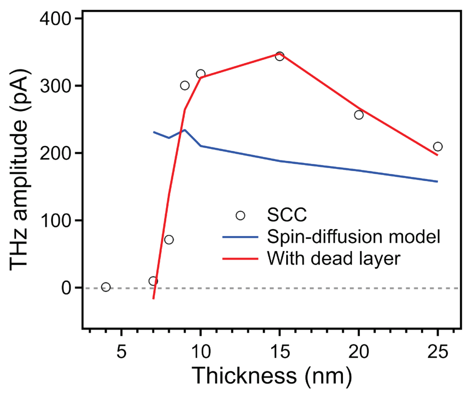

Figure S10. Spintronic THz amplitude as a function of the $Bi_{0.8}Sb_{0.2}$ thickness and fitting results with the spin-diffusion model.

We also measured THz transmittance and pump absorptance to obtain the impedance and absorptance of the Co/$Bi_{1-x}Sb_x$ (10 nm). As the Sb concentration increased, the impedance increased slightly and then decreased after $x = 0.07$, while the absorptance decreased monotonically, resulting in a decrease in the ultrafast spin current from the Co layer (**Figure S11**). Therefore, the rapidly increasing THz amplitude with Sb concentration for $0.04 \leq x \leq 0.2$ cannot be caused by the impedance and spin current based on the equation

$$S(\omega) \propto Z(\omega) \sum_i \int dz\ \gamma_i(z) j_s(z, \omega).$$

This implies that the spin Hall angle increased across the topological phase transition from trivial to nontrivial. Because atomic spin–orbit coupling of Bi is stronger than that of Sb, spin–orbit coupling strength decreased as the Sb concentration increased. Thus, it is natural to expect that the SCC of the bulk state is weakened in the nontrivial phase.[15,16] Therefore, we concluded that the enhancement of the SCC and spintronic THz emission is caused by the topological surface state. In addition, because the charge conductivity (σ_c) increases with Sb concentration, based on our results, the $Bi_{0.2}Sb_{0.8}$ film is the most promising for spin–orbit torque switching device whose power consumption is proportional to $1/(\sigma_c \gamma^2)$.

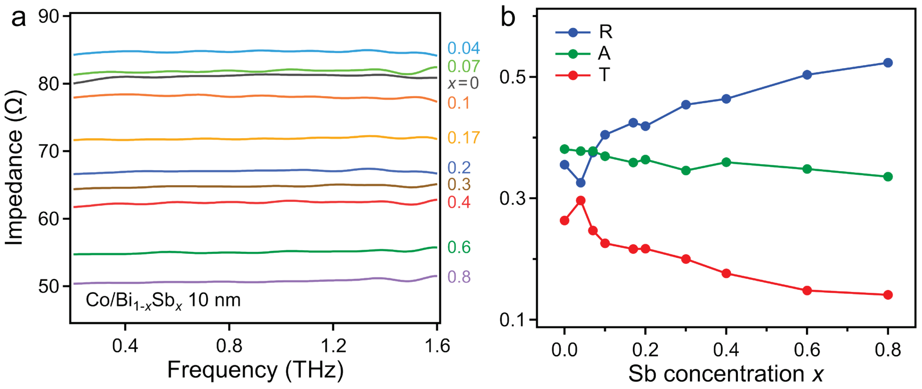

Figure S11. (a) Impedance of Co/$Bi_{1-x}Sb_x$ (10 nm). (b) Reflectance, absorptance, and transmittance of the Co/$Bi_{1-x}Sb_x$ (10 nm) as a function of the Sb concentration.

References

[1] H. Okamoto, *Journal of Phase Equilibria and Diffusion* **2012**, *33*, 493.
[2] S. Xiao, D. Wei, X. Jin, *Physical Review Letters* **2012**, *109*, 166805.
[3] J.-M. L. Beaujour, W. Chen, A. D. Kent, J. Z. Sun, *Journal of Applied Physics* **2006**, *99*, 08N503.

[4] X. Wang, L. Cheng, D. Zhu, Y. Wu, M. Chen, Y. Wang, D. Zhao, C. B. Boothroyd, Y. M. Lam, J.-X. Zhu, M. Battiato, J. C. W. Song, H. Yang, E. E. M. Chia, *Advanced Materials* **2018**, *30*, 1802356.
[5] Y. Huang, Z. Yao, C. He, L. Zhu, L. Zhang, J. Bai, X. Xu, *Journal of Physics: Condensed Matter* **2019**, *31*, 153001.
[6] Y. S. Lee, *Principles of Terahertz Science and Technology*, Springer US, Boston, MA, **2009**.
[7] Y. Wu, M. Elyasi, X. Qiu, M. Chen, Y. Liu, L. Ke, H. Yang, *Advanced Materials* **2017**, *29*, 1603031.
[8] H. Park, K. Jeong, I. Maeng, K. I. Sim, S. Pathak, J. Kim, S.-B. Hong, T. S. Jung, C. Kang, J. H. Kim, J. Hong, M. Cho, *ACS Applied Materials & Interfaces* **2021**, *13*, 23153.
[9] M. Tong, Y. Hu, Z. Wang, T. Zhou, X. Xie, X. Cheng, T. Jiang, *Nano Letters* **2021**, *21*, 60.
[10] T. Seifert, S. Jaiswal, U. Martens, J. Hannegan, L. Braun, P. Maldonado, F. Freimuth, A. Kronenberg, J. Henrizi, I. Radu, E. Beaurepaire, Y. Mokrousov, P. M. Oppeneer, M. Jourdan, G. Jakob, D. Turchinovich, L. M. Hayden, M. Wolf, M. Münzenberg, M. Kläui, T. Kampfrath, *Nature Photonics* **2016**, *10*, 483.
[11] O. Mosendz, J. E. Pearson, F. Y. Fradin, G. E. W. Bauer, S. D. Bader, A. Hoffmann, *Physical Review Letters* **2010**, *104*, 046601.
[12] H. Wang, J. Kally, J. S. Lee, T. Liu, H. Chang, D. R. Hickey, K. A. Mkhoyan, M. Wu, A. Richardella, N. Samarth, *Physical Review Letters* **2016**, *117*, 076601.
[13] D. Hou, Z. Qiu, K. Harii, Y. Kajiwara, K. Uchida, Y. Fujikawa, H. Nakayama, T. Yoshino, T. An, K. Ando, X. Jin, E. Saitoh, *Applied Physics Letters* **2012**, *101*, 042403.
[14] H. Emoto, Y. Ando, E. Shikoh, Y. Fuseya, T. Shinjo, M. Shiraishi, *Journal of Applied Physics* **2014**, *115*, 3.
[15] Z. Chi, Y.-C. Lau, X. Xu, T. Ohkubo, K. Hono, M. Hayashi, *Science Advances* **2020**, *6*, eaay2324.
[16] C. Şahin, M. E. Flatté, *Physical Review Letters* **2015**, *114*, 107201.
